# Zooming In: Assessing the Transferability of SDMs From Large to Small Spatial Extents

**DOI:** 10.1002/ece3.72419

**Published:** 2025-12-11

**Authors:** Jur R. G. Seuren, Martin C. E. Droog, Jelle P. Hilbers, Aafke M. Schipper

**Affiliations:** ^1^ Department of Environmental Science, Radboud Institute for Biological and Environmental Sciences (RIBES) Radboud University Nijmegen the Netherlands; ^2^ Haskoning Nederland B.V. Amersfoort the Netherlands; ^3^ PBL Netherlands Environmental Assessment Agency The Hague the Netherlands

**Keywords:** biodiversity, ensemble SDMs, model accuracy, model performance, reptiles, transferability

## Abstract

Species Distribution Models (SDMs) represent a common method for predicting biodiversity responses to environmental changes. SDMs are typically established for and applied to large areas, while little is known about the transferability of large‐scale SDMs to smaller spatial extents. This study aims to fill this gap by fitting country‐level ensemble SDMs for the seven native reptile species occurring in the Netherlands, and testing their predictive ability against independent occurrence data in 10 municipalities. For each of the species, we trained an ensemble SDM using bioclimatic variables, land cover fractions and soil type as predictors. We then evaluated model performance in 10 municipalities that were not included in model training. Despite performing well at national extent, with a mean cross‐validated True Skill Statistic (TSS) value of 0.85 (range of 0.76–0.94) across the species, the ensemble SDMs generally yielded poor to moderate performance scores for the municipalities. Averaged per species, TSS values ranged from 0.05 to 0.57, while mean TSS values per municipality ranged from −0.07 to 0.61. The general decrease in model performance from national to local extent was mainly due to a decline in model specificity (i.e., the ability to predict absence). A two‐way ANOVA showed model performance varied significantly among both municipalities and species (*p* < 0.001). Our results suggest that while ensemble SDMs fitted across large spatial extents can provide a rough indication of the potential distribution of a species, caution should be applied when informing local conservation decisions based on broad‐scale SDM outcomes.

## Graphical abstract

In this study, we trained ensemble SDMs for the seven native reptile species occurring in the Netherlands, and tested their predictive ability against independent occurrence data in 10 municipalities. We found that despite performing well at national extent, the ensemble SDMs showed poor to moderate performance at municipal level. Our results suggest that caution is needed when using large‐scale SDMs to inform local conservation decisions.

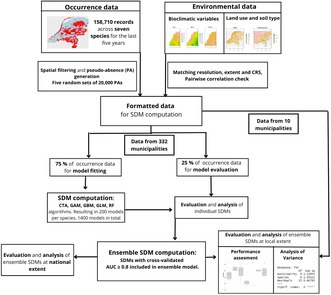

## 1 Introduction

The world is currently in an era of unprecedented changes. The global climate is changing due to anthropogenic emissions of greenhouse gases (Masson‐Delmotte et al. 2021), which have culminated in a global mean temperature increase of around 1.1°C compared to pre‐industrial times and an increase in the frequency and magnitude of extreme weather events (Lee et al. 2023; Stott 2016). At the same time, conversion of natural vegetation for agriculture and expansion of human settlements are causing habitat loss, degradation and fragmentation (Theodorou 2022). The changes in climate and expansion of land use are among the main direct drivers of the current global biodiversity crisis (Jaureguiberry et al. 2022). Understanding how biodiversity responds to ongoing and expected future global environmental change is imperative for designing effective biodiversity conservation measures (Raymond et al. 2022).

Species Distribution Models (SDMs) are commonly used to assess the influence of large‐scale environmental changes on biodiversity (Hao et al. 2019; Zurell et al. 2020). SDMs are correlative models that relate point occurrence records of a species to a selection of environmental variables, typically including variables representative of climate and land use (Miller 2010). SDMs can be used to estimate species‐environment relationships, map a given species' geographical distribution, and predict the effects of changes in environmental conditions on the distribution of that species. SDMs can be applied in, for example, conservation prioritisation, protected area design, invasive species management and climate change impact predictions (Villero et al. 2017; Ferrari et al. 2018). Yet, while SDMs are standard practice in especially climate change impact studies, concerns have been raised about their accuracy (Rodríguez‐Rey et al. 2013; Santini et al. 2021). For example, SDMs trained with a small number of species observations, including irrelevant predictor variables or ignoring relevant ecological processes (e.g., dispersal constraints) have been shown to yield inaccurate predictions of species' occurrences (Dormann et al. 2018; Soultan and Safi 2017). Hence, insight into the performance of SDMs related to various methodological choices made when building and applying these models is vital in view of their supporting role in numerous aspects of nature conservation. Recent years have seen a surge in research that explores different aspects of SDMs, leading for instance to recommendations for best practices (Araújo et al. 2019). Nevertheless, important knowledge gaps remain.

Among the less‐studied aspects of SDMs is their transferability across spatial extents. While the majority of studies train and apply SDMs at relatively large spatial extents (national, continental or even global extent), much less is known about the performance of SDMs trained at a large extent, and subsequently applied to a much smaller region (Austin and Van Niel 2011). Nonetheless, this application could hold significant value for supporting local‐level biodiversity policy, for example by assessing the potential effects of local land cover changes or the implementation of biodiversity‐friendly management measures (Gogol‐Prokurat 2011; Mateo et al. 2019). Existing studies show that the transferability of SDMs depends on the assessed species' traits and range, geographical characteristics of the distribution, model complexity and choice of predictor variables (Werkowska et al. 2017; Rousseau and Betts 2022). Such dependencies illustrate the need for further research to identify potential generalities in the transferability of SDMs.

This study aims to contribute to knowledge on SDM transferability across spatial extents by assessing the performance of ensemble SDMs calibrated at a national level when applied to municipalities. To this end, we trained ensemble SDMs for the seven native reptile species occurring in the Netherlands, and tested their predictions against independent occurrence data in 10 municipalities. The Netherlands is strongly impacted by climate change; temperatures have increased by twice the global average temperature rise, while extreme meteorological events are increasing in intensity and frequency (Junk et al. 2019; Compendium voor de Leefomgeving 2020). Furthermore, the Netherlands is one of the most densely populated countries globally, with remaining patches of natural areas being of a highly fragmented nature (Bergers and Kalkhoven 1996). As reptiles are highly sensitive to the effects of both climate and land use change, their numbers in the Netherlands have strongly declined and six out of seven of the native reptile species are currently on the Dutch Red List (Böhm et al. 2016; Creemers et al. 2023). As knowledge on reptiles' responses to environmental changes is vital in the light of their conservation, accurate ensemble SDMs could prove valuable in support of their protection in the face of environmental change.

## 2 Methodology

### 2.1 Species Occurrence Data

We downloaded geo‐referenced occurrence data of the seven reptile species native to the Netherlands from the National Database Flora and Fauna (NDFF) in March 2024. The occurrence records in this database are either collected according to a standardised protocol (14%) or subjected to expert validation (86%), yielding accurate geo‐referenced occurrence data (Kosmala et al. 2016). For this study, we extracted the maximum number of occurrence records tagged as being ‘reliable’ from the period 2019–2024, to which we had access with our NDFF subscription. The number of occurrence records varied from 5439 for smooth snake to 47,040 for sand lizard (Table S1).

We converted the coordinates of the occurrence records, which were in a national projection, to WGS84 in order to match the coordinate reference system of the environmental variable data (see below). Further, we sub‐sampled one record per 250 m grid cell in order to avoid pseudo‐replication (Boria et al. 2014). Given that the occurrence data were presence‐only, we generated pseudo‐absences (PAs) prior to constructing the SDMs. For each species, we generated five sets of 20,000 pseudo‐absences by randomly selecting cells without presence records, following recommendations from previous work which showed that generating a large number of PAs and several PA sets tends to result in better SDM performance (Barbet‐Massin et al. 2012). We selected no more than one PA per grid cell.

### 2.2 Environmental Variables

We used a combination of climate, land use, and soil type variables to model species distributions. We selected four bioclimatic variables from the ‘bioclim’ database (Fick and Hijmans 2017), which provides 19 biologically relevant climatic variables deduced from monthly precipitation and temperature values in the WorldClim database. These data are available at a resolution of 30 arc seconds, corresponding with ~1 km. Our selection included mean annual temperature, temperature seasonality, mean annual precipitation and precipitation seasonality (BIO1, BIO4, BIO12 and BIO15). This selection of climatological variables captures the main characteristics of a location's climate and is commonly used in SDMs (Austin and Van Niel 2011; Čengić et al. 2020; Falaschi et al. 2019). Additionally, we incorporated land use characteristics and soil properties, which were previously found to be important determinants of reptile occurrence (Cordier et al. 2021; Harings et al. 2014; Jácome‐Flores et al. 2015). The Land Use Database of the Netherlands classifies land use in the Netherlands into 52 categories at a spatial resolution of 25 m (Hazeu et al. 2023). We obtained data on soil type from the Landscape Soil Map 2023 which classifies soil types in the Netherlands into 113 categories at a spatial resolution of 250 m (Van Delft and Maas 2023).

To ensure compatibility, we matched the resolution and extent of all environmental variables, taking the soil type dataset (250 m) as a basis. We upscaled the land use data by obtaining the cover fractions of the original land use categories in the new, larger cell. Based on vegetation structure, we generalised the 52 categories of land use to 27 categories (Table S2). Similarly, we reclassified soil type to 18 categories based on generalising the initial classification (Table S3). We downscaled the climatological data to a resolution of 250 m using bilinear interpolation, which is a common approach for continuous data (Fick and Hijmans 2017; Latombe et al. 2018). Finally, we checked for collinearity among the climatological variables and the land use fractions by computing a pairwise correlation matrix based on Pearson's correlation coefficient, to prevent including highly correlated variables in the same models. We preferred a bivariate correlation analysis over a multicollinearity assessment based on variance inflation factors (VIFs), because the former better facilitates the selection of the ecologically more relevant variables to be retained. We set the threshold for Pearson's r at 0.8 (Valavi et al. 2022). All pairwise correlations remained below this threshold.

### 2.3 Setting Aside Test Data

**FIGURE 1:**
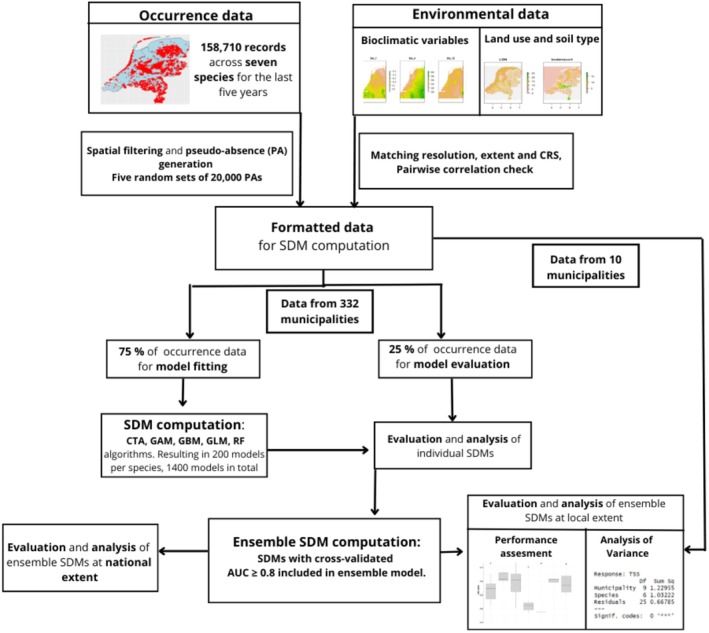
Overview of the main methodological steps taken in this study. We refer to the methodology description for details.

To be able to assess the performance of the SDMs at the level of municipalities, we excluded environmental variables, presence and (pseudo‐)absence data for 10 Dutch municipalities from model training (Figure 1). To this end, we first assigned each record to a municipality based on its coordinates and a map with municipality borders using ArcGIS Pro (version 3.2.1) (Centraal Bureau voor de Statistiek 2023). We then set aside data from 10 municipalities for model testing, and retained the other municipalities for model training (see Section 2.4). We selected the 10 municipalities based on geographic spread, ensuring that they represent a range of climatic conditions, soil types and land uses in the Netherlands (Figure S1), and such that each of them contained occurrence records for at least one of the seven reptile species. The number of reptile species observed across the municipalities in our test dataset ranged from one for Wormerland to all seven for Westerveld (Table S4). We used the data for these areas to test the SDM predictions against independent occurrence data at the much smaller spatial extent of municipalities.

### 2.4 Constructing the Ensemble SDMs

Using the ‘biomod2’ package (Thuiller et al. 2016) in R (version 4.3.3), we established an ensemble SDMs for each species based on five modelling techniques: Classification Tree Analysis (CTA), Generalised Additive Model (GAM), Generalised Linear Model (GLM), Gradient Boosting Machine (GBM) and Random Forest (RF). This selection covers three major categories of modelling approaches (regression, machine learning, and classification‐based) and consists of techniques that are commonly used in SDM studies (Čengić et al. 2020; Duan et al. 2014; Hao et al. 2019; Marmion et al. 2009). For each species, we computed the importance of the environmental variables using the ‘bm_VariablesImportance’ function included in ‘biomod2’ (Table S5). In the model fitting, we ensured that pseudo‐absences were weighted equally compared to presence records (Barbet‐Massin et al. 2012). Further, we employed an eight‐fold cross‐validation by splitting the training data into four equal subsets, using three subsets (75% of the data) for model training and the remainder (25%) for evaluation (Guillaumot et al. 2019; Hao et al. 2020; Zhang et al. 2020). The dataset used for determining model performance alternated between the four data subsets for each of the eight cross‐validation replicates, yielding a full replication of the cross‐validation cycle, within which each data subset, containing 25% of the occurrence data, was used twice to determine model performance.

We combined the models obtained (200 per species, based on five modelling techniques, five PA sets, and eight cross‐validation runs) into one ensemble prediction for every species. Models were included in the ensemble model if they exceeded a threshold of 0.8 for the Area under the Receiver‐Operating Characteristic Curve (AUC) metric, following common practice (Hao et al. 2019; Ramirez‐Reyes et al. 2021). Based on AUC values, we assigned better performing models proportionally more weight within the ensemble models (Marmion et al. 2009). Next, we binarized the ensemble SDMs based on a threshold for the probability of occurrence that maximised the True Skill Statistic (TSS) value of the ensemble model. We evaluated the performance of all resulting ensemble models using the AUC, TSS, sensitivity and specificity metrics (Allouche et al. 2006).

### 2.5 Evaluating Transferability

We evaluated model performance per species and per municipality based on TSS as well as sensitivity and specificity scores. Because not all seven species occur in every municipality (Table S4), this yielded a total of 38 species‐municipality combinations with corresponding sets of model performance scores. Next, we carried out a two‐way analysis of variance (ANOVA) to elucidate the importance of the identity of the species and municipalities for the observed variability of TSS values. Based on the output of the two‐way ANOVA, we computed the *η* ^2^ value to explore how much of the variation in TSS scores was explained by species and municipality, respectively (Adams and Conway 2021). We calculated the *η* ^2^ value by dividing the sum of squares for species and municipality by the total sum of squares. We performed these analyses using the ‘stats’ package in R (R Core Team 2022).

## 3 Results

**FIGURE 2:**
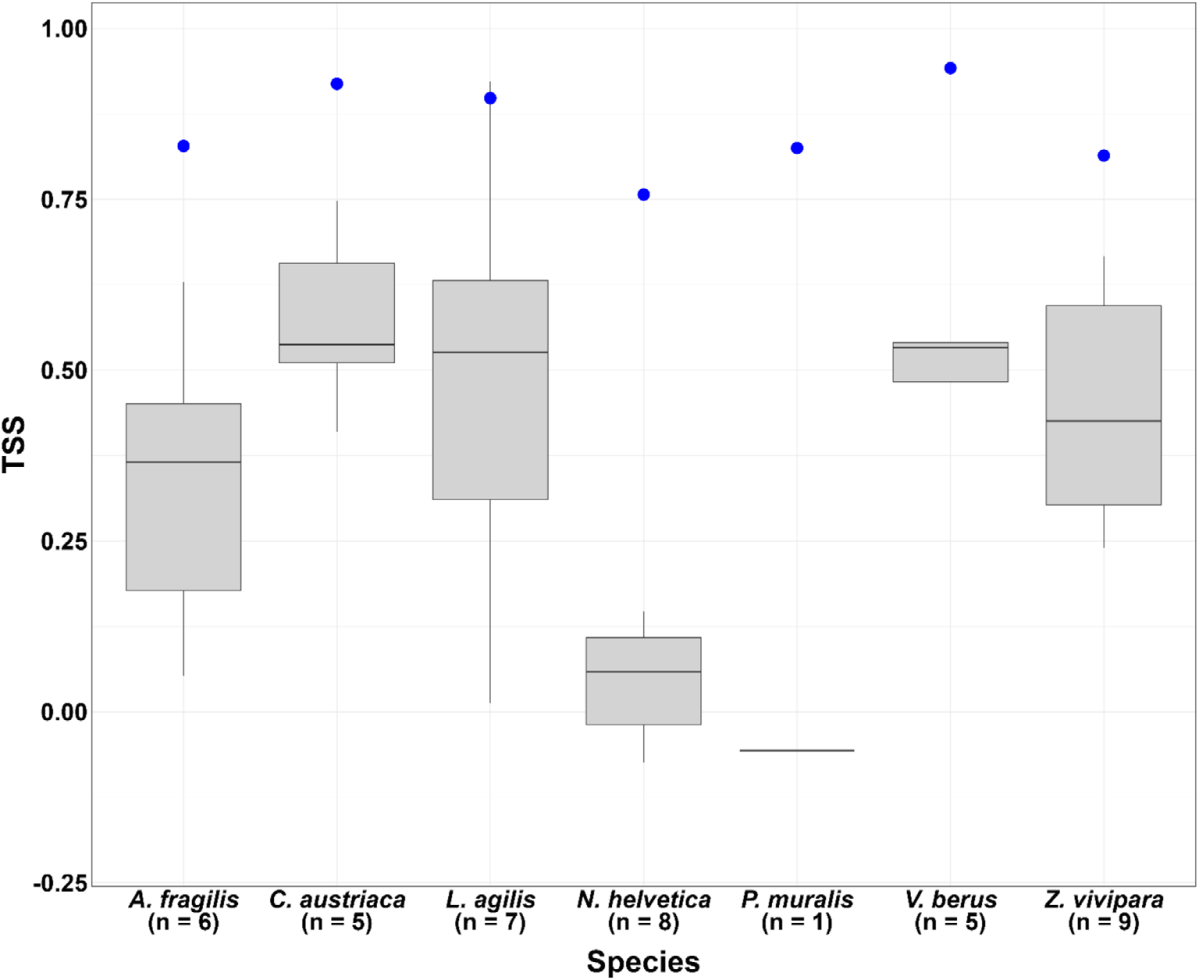
TSS scores per species of ensemble SDMs trained at the extent of the country when applied to the municipal level. Median values are displayed by the horizontal black line within the boxes showing the interquartile range (IQR) (distance between first and third quantiles of data). Whiskers extend to the points lying at most 1.5 times IQR‐distance from the edge of the upper and lower limits of the box. Country‐level TSS values for the trained ensemble models are provided for comparison (blue dots). Sample sizes (*x*‐axis) represent the number of municipalities used in the evaluation (Table S5).

**FIGURE 3:**
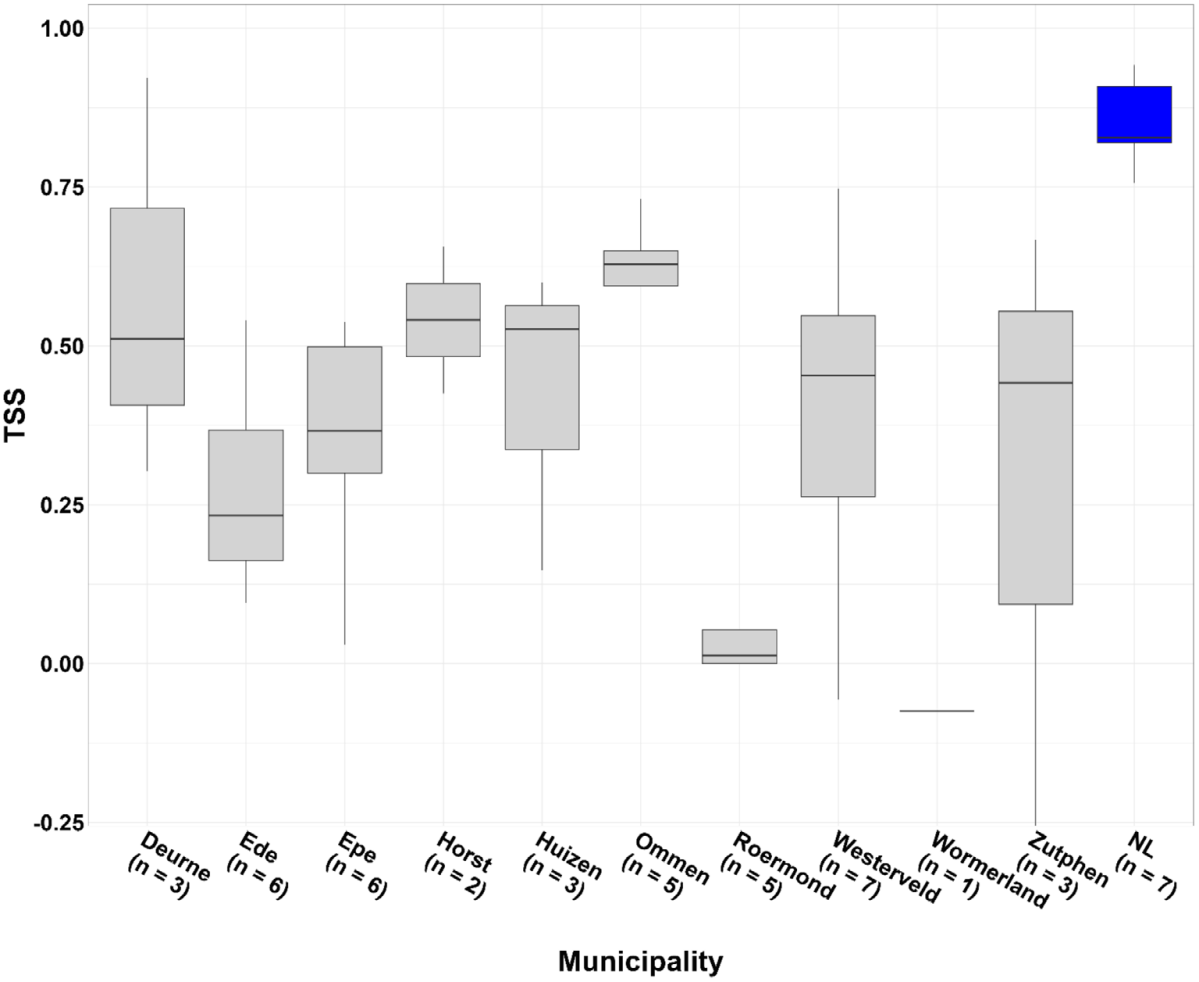
TSS scores per municipality of ensemble SDMs trained at the extent of the country when applied to the municipal level. Median values are displayed by the horizontal black line within the boxes showing the interquartile range (IQR) (distance between first and third quantiles of data). Whiskers extend to the points lying at most 1.5 times IQR‐distance from the edge of the upper and lower limits of the box. Country‐level TSS values for the trained ensemble models are provided for comparison (blue boxplot). Sample sizes (*x*‐axis) represent the number of species used in the evaluation (Table S5).

While the SDMs showed high performance based on the training data, with cross‐validated TSS values ranging from 0.70 for *N. helvetica* to 0.94 for *V. berus* (Table S6), we found considerably lower performance in the application to the 10 municipalities. Per species, mean TSS scores across the municipalities ranged from −0.05 (SD: 0.00, *n* = 1) for *P. muralis* to 0.57 (SD: 0.13, *n* = 5) for *C. austriaca* (Figure 2). Aggregated across species per municipality, TSS values ranged from −0.07 (SD: 0.00, *n* = 1) for Wormerland to 0.61 (SD: 0.10, *n* = 5) for Ommen (Figure 3).

In general, we found model sensitivity values to be higher and less variable than model specificity (Figures S2 and S3). Clear exceptions to this pattern were the species *P. muralis* and the municipality Wormerland, which scored much higher for model specificity. It should be noted that these two results are based on a sample size of only 1.

The two‐way ANOVA showed that both species (*F* = 6.4400, df = 6, *p* < 0.001) and municipality (*F* = 5.1141, df = 9, *p* < 0.001) explained significant shares of the variance in TSS scores. Municipality explained slightly more of the variance in TSS scores (*η* ^2^ = 0.42) than species (*η* ^2^ = 0.35). These two variables together accounted for 77% of total observed variance in TSS values.

## 4 Discussion

The main aim of this study was to assess the performance of ensemble SDMs fitted at the national level when applied to a local level, and identify whether national‐level SDMs can be used to inform local biodiversity management. While we found good to excellent performance for the ensemble SDMs at the national level, with cross‐validated TSS values of at least 0.75 for all seven reptile species, performance was much lower at the level of the municipalities. For most of the species, this was due to the relatively low specificity of the SDMs, reflecting a large proportion of false positives, indicating overprediction of local species' distributions by our national‐level ensemble SDMs. The sensitivity and specificity values also indicate that the ensemble SDMs more accurately predict presences than absences at the local level, while no such difference was observed at the national level (Figures S2 and S3, Table S5). This illustrates the main take‐away of this study: while the national‐level ensemble SDMs might be able to predict the broad‐scale potential distributions of the species within the Netherlands, they seem to fall short in distinguishing between suitable and unsuitable habitat patches at the local level. We also observed reductions in other performance metrics (Table S7).

There are two reasons that might explain the lack of transferability of the ensemble SDMs to local extents. A first potential explanation for the inaccurate ensemble SDM predictions at the local level is our method of selecting individual SDMs for the ensemble models. We selected models if their AUC score exceeded 0.80, representing a common approach (Hao et al. 2019). This resulted in machine learning‐based individual SDMs (particularly the model obtained with the RF algorithm) being assigned a lot of weight in the ensemble models, as their AUC scores were often exceptionally high. Contrarily, regression‐based models (GLMs) displayed lower AUC scores and were thus assigned less weight during the ensemble SDM computation. However, the environmental response curves obtained by the GLM and RF techniques indicate that the AUC‐driven computation of ensemble SDMs does not necessarily lead to the most ecologically plausible individual SDMs being selected (Figure S4). For example, for *C. austriaca* , the RF technique resulted in ragged response curves, predicting habitat suitability to be either high or low, with sharp transitions between the two, based on the value of the predictor (Figure S4). This suggests that this modelling technique is prone to overfitting (Merow et al. 2014). The GLM, on the other hand, revealed (parts of) the typical bell‐shaped response curves one would expect a species to show in response to gradients of abiotic variables (Westman 1980). Therefore, it could be argued that for the sake of ecological relevancy and transferability to other conditions and locations, it might actually be more pertinent to allocate more weight to the GLM in the ensemble SDMs, despite lower predictive performance (Merow et al. 2014). The larger weights assigned to (overfitted) models might have contributed to inaccurate ensemble SDM predictions at the municipal level, illustrating the necessity to look beyond statistics and retain a critical attitude regarding the ecological plausibility of SDMs (Hellegers et al. 2020).

A second possible explanation for the drop in performance from national to local extent relates to the properties of TSS as a performance metric. Specifically, recent evidence suggests that TSS favours correctly predicted presences over correctly predicted absences when the number of presences is low relative to the number of absences (Leroy et al. 2018; Hellegers et al. 2025). With a large number of absences, as in our model training dataset at national extent, the number of true negatives is exceptionally high, leading to high specificity (see Table S5) even if there is a considerable number of false positives. Thus, false positives are penalised less than false negatives, leading to high TSS values even when the distribution of the species is overpredicted (Leroy et al. 2018; Hellegers et al. 2025). However, when the number of true absences is much lower, as in our test municipalities, the false positives become more decisive for the specificity, resulting in a considerable drop in specificity hence TSS, while sensitivity remains high (compare Figures S2 and S3, Table S5). These intricacies of TSS indicate that high TSS values might be misleading and point at the need to consider sensitivity and specificity also separately or, additionally, other performance metrics alongside TSS. Further, it might be appropriate to refrain from translating predicted probabilities of occurrence or habitat suitability scores into binary presence–absence predictions when employing broad‐scale SDMs in local applications.

To summarise, the results of this study show a relatively low transferability of broad‐scale (ensemble) SDMs to local extents. This supports existing literature in highlighting the challenging nature of transferring SDMs across space (Rousseau and Betts 2022; Werkowska et al. 2017). Our results might partly reflect model overfitting, while the choice of TSS as a performance metric might also contribute to the perceived lack of transferability of our ensemble SDMs. Furthermore, inherent characteristics of (ensemble) SDMs, such as their simple, correlative nature, lacking, for example, biotic interactions, and the relatively small set of environmental predictor variables could also constrain performance at local extents (Dormann et al. 2012; Evans et al. 2015; Scherrer and Guisan 2019). Finally, the 250 m resolution of input data used in this study might be insufficient to capture the fine‐grain environmental heterogeneity that is particularly relevant at local extents, potentially allowing important relationships to go unnoticed in the resulting ensemble SDMs (Waldner et al. 2018; Sinclair et al. 2010). These factors might contribute to the SDMs failing to capture local‐scale variation in habitat suitability, even though the models can predict the broader potential distribution or large‐scale habitat suitability of a species reasonably well.

Our results show that the performance of national‐level ensemble SDMs can be highly variable and generally classifies as poor to moderate in local applications. Though the ensemble SDMs correctly predicted the majority of species' presences at a municipal extent, the proportion of false positives was substantial. This suggests that the national‐level SDMs can be used to obtain a first indication of the potential distributions of the species and the main factors determining its range, but that additional field observations, more fine‐grained models or a combination of coarse and fine‐grain models in so‐called nested SDMs are needed to underpin actual conservation decisions (Guisan et al. 2025). Follow‐up studies exploring different ensemble modelling selection methods, performance metrics, a higher input data resolution and/or a nested or mechanistic modelling approach could generate valuable additional insights into the transferability of SDMs across spatial extents and its underlying factors. Based on our results, we would advise against basing local biodiversity policy on predictions of SDMs trained at a much larger spatial extent. However, our results revealed good to excellent model performance at the national level, suggesting that (ensemble) SDMs could be valuable for informing nature conservation policies at larger spatial extents and for distilling coarse‐grain habitat requirements.

## Author Contributions

**Jur R. G. Seuren:** conceptualization (equal), data curation (lead), formal analysis (lead), methodology (equal), software (lead), visualization (lead), writing – original draft (lead), writing – review and editing (equal). **Martin C. E. Droog:** conceptualization (equal), methodology (equal), software (supporting), supervision (equal), writing – review and editing (equal). **Jelle P. Hilbers:** methodology (supporting), software (supporting), supervision (equal), writing – review and editing (equal). **Aafke M. Schipper:** conceptualization (equal), methodology (supporting), supervision (equal), writing – original draft (supporting), writing – review and editing (equal).

## Conflicts of Interest

The authors declare no conflicts of interest.

## Supporting information


**Appendix S1:** ece372419‐sup‐0001‐Supinfo.docx.

## Data Availability

The data and code used to generate the findings and figures presented in this study are available at Zenodo at https://doi.org/10.5281/zenodo.17017856. The occurrence data used to generate the SDMs includes geo‐referenced data of sensitive and protected species, and as such has been aggregated to a resolution of 5 km to comply with the guidelines of the National Database Flora and Fauna (NDFF).
