# Supplementary material for: Zooming In: Assessing the Transferability of SDMs From Large to Small Spatial Extents
Source: Ecol Evol. 2025 Dec 11;15(12):e72419. doi: 10.1002/ece3.72419 (PMC12696477; doi:10.1002/ece3.72419)
Supplement: Supplementary file 1 — Appendix S1: ece372419‐sup‐0001‐Supinfo.docx. [file ECE3-15-e72419-s001.docx]

**Supplementary Information for:**

**Zooming in: assessing the transferability of SDMs from large to small spatial extents**

**Transferability of SDMs across extents**

**Table of contents**

[Tables 2](#_Toc209001110)

[Table S1 2](#_Toc209001111)

[Table S2 2](#_Toc209001112)

[Table S3 3](#_Toc209001113)

[Table S4 8](#_Toc209001114)

[Table S5 9](#_Toc209001115)

[Table S6 9](#_Toc209001116)

[Table S7 10](#_Toc209001117)

[Figures 11](#_Toc209001118)

[Figure S1 11](#_Toc209001119)

[Figure S2 12](#_Toc209001120)

[Figure S3 12](#_Toc209001121)

[Figure S4 13](#_Toc209001122)

# **Tables**

**Table S1 | Overview of the number of occurrence records per species available for this study.**

| **Species** | **Number of occurrence records** |
| --- | --- |
| European viper (*Vipera berus*) | 13231 |
| Smooth snake (*Coronella austriaca*) | 5439 |
| Slow worm (*Anguis fragilis*) | 17535 |
| Viviparous lizard (*Zootoca vivipara*) | 42724 |
| Common wall lizard (*Podarcis muralis*) | 10275 |
| Grass snake (*Natrix helvetica*) | 22446 |
| Sand lizard (*Lacerta agilis*) | 47040 |

**Table S2 | Reclassification of land use categories and codes obtained from the Dutch Land Use database.**

| **Original**  **code** | **New**  **code** | **Original category** | **New category** |
| --- | --- | --- | --- |
| 1 | 1 | Agricultural grassland | Grassland |
| 2 | 2 | Corn | Agriculture |
| 3 | 2 | Potatoes | Agriculture |
| 4 | 2 | Beets | Agriculture |
| 5 | 2 | Grains | Agriculture |
| 6 | 2 | Other crops | Agriculture |
| 8 | 3 | Greenhouses | Greenhouses |
| 9 | 4 | Orchard | Orchard |
| 10 | 2 | Flower bulbs | Agriculture |
| 11 | 5 | Deciduous forest | Deciduous forest |
| 12 | 6 | Coniferous forest | Coniferous forest |
| 16 | 7 | Freshwater | Freshwater |
| 17 | 8 | Salt water | Salt water |
| 18 | 9 | Building in primary agglomeration | Built area |
| 19 | 9 | Building in secondary agglomeration | Built area |
| 20 | 10 | Forest in primary agglomeration | Forest in built area |
| 22 | 10 | Forest in secondary agglomeration | Forest in built area |
| 23 | 11 | Grass in primary agglomeration | Grass in built area |
| 24 | 11 | Barren ground in agglomeration | Grass in built area |
| 26 | 9 | Building in rural area | Built area |
| 27 | 9 | Other land use in rural area | Built area |
| 28 | 11 | Grass in secondary agglomeration | Grass in built area |
| 29 | 12 | Solar park | Paved area |
| 30 | 13 | Salt marsh | Marsh vegetation |
| 31 | 15 | Open sands in coastal area | Open sands |
| 32 | 15 | Dunes (low vegetation) | Dune |
| 33 | 15 | Dunes (high vegetation) | Dune |
| 34 | 16 | Dune heather | Heather |
| 35 | 14 | Open sands/riverine sand deposits | Open sands |
| 36 | 16 | Heather | Heather |
| 37 | 17 | Moderately grass encroached heather | Moderately grass encroached heather |
| 38 | 18 | Strongly grass encroached heather | Strongly grass encroached heather |
| 39 | 19 | Raised bog | Raised bog |
| 40 | 20 | Forest in raised bog area | Forest in raised bog area |
| 41 | 13 | Other marsh vegetation | Marsh vegetation |
| 42 | 21 | Reeds | Reed vegetation |
| 43 | 22 | Forest in marsh area | Forest in marsh area |
| 45 | 1 | Naturally managed agricultural grassland | Grassland |
| 46 | 23 | Coastal grassland | Grassland (coastal area) |
| 47 | 1 | Other grass | Grassland |
| 61 | 4 | Tree orchards | Orchard |
| 62 | 4 | Fruit orchards | Orchard |
| 251 | 12 | Main infrastructure and railways | Paved area |
| 252 | 12 | Semi-paved roads, slow traffic  infrastructure and other infrastructure | Paved area |
| 253 | 12 | Narrow roads | Paved area |
| 321 | 24 | Shrub vegetation in raised bog (low) | Shrub vegetation in raised bog |
| 322 | 25 | Shrub vegetation in marsh (low) | Shrub vegetation in marsh |
| 323 | 26 | Other shrub vegetation (low) | Other shrub vegetation |
| 331 | 24 | Shrub vegetation in raised bog (high) | Shrub vegetation in raised bog |
| 332 | 25 | Shrub vegetation in marsh (high) | Shrub vegetation in marsh |
| 333 | 26 | Other shrub vegetation (high) | Other shrub vegetation |
| 0 | 0 | No data | No data |

**Table S3 | Reclassification of soil type categories and codes in the Landscape Soil Map 2023 database to more general categories.**

| **Original**  **code** | **New**  **code** | **Original category** | **New category** |
| --- | --- | --- | --- |
| 1 | 2 | Sandy soils, rich in minerals | Calcareous sandy soil |
| 2 | 2 | River dunes, sandy levees and calcareous Pleistocene sand deposits | Calcareous sandy soil |
| 3 | 4 | River dunes, sandy levees and non-calcareous Pleistocene sand deposits | Non-calcareous sandy soil |
| 4 | 4 | Sandy soils poor in nutrients and bases | Non-calcareous sandy soil |
| 5 | 2 | High sandy soils with black agricultural cover | Calcareous sandy soil |
| 6 | 2 | High sandy soils with brown agricultural cover | Calcareous sandy soil |
| 7 | 2 | Initial moisture- and base-retaining sandy soil | Calcareous sandy soil |
| 8 | 2 | Initial moisture- and base-retaining sandy soil, rich in iron | Calcareous sandy soil |
| 9 | 4 | Sandy soils poor in bases with laterally intruding calcium-poor groundwater | Non-calcareous sandy soil |
| 10 | 4 | Moist sandy soils poor in nutrients and bases | Non-calcareous sandy soil |
| 11 | 2 | Sandy soils fed by weak seepage | Calcareous sandy soil |
| 12 | 2 | Sandy soils fed by weak seepage, rich in iron | Calcareous sandy soil |
| 13 | 2 | Humic sandy soil with seepage | Calcareous sandy soil |
| 16 | 2 | Calcareous fine-grained riverine sandy soil | Non-calcareous sandy soil |
| 17 | 4 | Non-calcareous fine-grained riverine sandy soil | Non-calcareous sandy soil |
| 18 | 4 | Non-calcareous fine-grained riverine sandy soil, rich in iron | Non-calcareous sandy soil |
| 19 | 2 | Sandy soils with calcareous arable land cover | Calcareous sandy soil |
| 21 | 4 | Sandy soils with stagnating rainwater, poor in nutrients and bases | Non-calcareous sandy soil |
| 22 | 2 | Sandy soils fed by localized seepage over loamy layer | Calcareous sandy soil |
| 23 | 4 | Peat extraction filled with sand | Non-calcareous sandy soil |
| 24 | 8 | Peat soil with moderate regional seepage, with sand cover | Peat soil |
| 25 | 8 | Peat soil with moderate regional seepage, rich in iron and with sand cover | Peat soil |
| 26 | 4 | Initial dry, base-poor sandy soil | Non-calcareous sandy soil |
| 27 | 4 | Initial moisture-retaining base poor sandy soil | Non-calcareous sandy soil |
| 28 | 2 | Calcareous, humus poor to moderately humic dry dunes | Calcareous sandy soil |
| 29 | 2 | Calcareous very humus poor dry dunes | Calcareous sandy soil |
| 32 | 4 | Descaled dry dune soil poor in bases | Non-calcareous sandy soil |
| 33 | 2 | Calcareous dune valleys or seepage lakes | Calcareous sandy soil |
| 34 | 2 | Silty, wet sandy soils | Calcareous sandy soil |
| 35 | 2 | Seepage fed sandy dune soils | Calcareous sandy soil |
| 36 | 2 | Sandflats and plains flooded daily (marine) | Calcareous sandy soil |
| 37 | 2 | Freshwater creeks, levees and bowls flooded daily | Calcareous sandy soil |
| 38 | 2 | Calcareous, cut-off beach plains and creek ridges | Calcareous sandy soil |
| 39 | 4 | Non-calcareous, cut-off beach plains and creek ridges | Non-calcareous sandy soil |
| 40 | 2 | Calcareous coarse-grained riverine sandy soil | Calcareous sandy soil |
| 41 | 4 | Non-calcareous coarse-grained riverine sandy soil | Non-calcareous sandy soil |
| 42 | 15 | Dry loess-loam soils | Loess soil |
| 43 | 11 | Terrace with sandy loam soil, clear hydromorphic characteristics absent | Loam soil |
| 44 | 11 | Terrace with silty loam soil, clear hydromorphic characteristics absent | Loam soil |
| 46 | 17 | Dry brook-valley bottoms | Dry valley bottom soil |
| 47 | 15 | Non-calcareous loess slopes | Loess soil |
| 48 | 4 | Slopes with non-calcareous slope material or solifluction cover | Non-calcareous sandy soil |
| 50 | 10 | Moist low-lying area | Calcareous clay soil |
| 52 | 11 | Terrace with strongly stagnating loamy soil | Loam soil |
| 53 | 15 | Slopes with cover of flushed loess on terrace material | Loess soil |
| 54 | 15 | Terrace with loess cover on non-flushed terrace material | Loess soil |
| 55 | 11 | Low-lying loam soil | Loam soil |
| 56 | 11 | Low lying loam soil rich in iron | Loam soil |
| 57 | 10 | Seepage zones and springs | Calcareous clay soil |
| 58 | 10 | Seepage fed ‘bowl’ soils | Calcareous clay soil |
| 59 | 15 | Terrace with loess cover on flint eluvium | Loess soil |
| 61 | 15 | Terrace or slope with flushed loess cover on marl | Loess soil |
| 62 | 15 | Terrace or slope with lime-poor slope material or solifluction cover on shallow marl | Loess soil |
| 63 | 11 | Intermittently moist clay-loam soil rich in minerals | Loam soil |
| 66 | 10 | Terrace remains in glauconite clay | Calcareous clay soil |
| 67 | 12 | Moisture retaining tertiary (or older) clay soil | Non-calcareous clay soil |
| 68 | 12 | Intermittently moist tertiary clay soil | Non-calcareous clay soil |
| 69 | 10 | Calcareous higher floodplains and ridges (clay) | Calcareous clay soil |
| 70 | 12 | Non- calcareous higher floodplains and ridges (clay) | Non-calcareous clay soil |
| 71 | 2 | Calcareous small levees and river ridges | Dikes and levees |
| 72 | 4 | Non-calcareous small levees and river ridges | Dikes and levees |
| 73 | 4 | Non-calcareous small levees and river ridges rich in iron | Dikes and levees |
| 74 | 7 | Calcareous clay soils | Calcareous clay soil |
| 75 | 10 | Calcareous ‘bowl soils’ | Calcareous clay soil |
| 76 | 12 | Non-calcareous ‘bowl soils’ | Non-calcareous clay soil |
| 77 | 10 | Calcareous sabulous and light clay soil within dike ring | Calcareous sabulous clay soil |
| 78 | 12 | Calcareous sabulous and light clay soils with sand in the upper 120 cm within dike ring | Calcareous sabulous clay soil |
| 80 | 14 | Non-calcareous sabulous and light clay soil within dike ring | Non-calcareous sabulous clay soil |
| 81 | 14 | Non-calcareous sabulous and light clay soils rich in iron with sand in the upper 120 cm within dike ring | Non-calcareous sabulous clay soil |
| 82 | 13 | Non-calcareous sabulous and light clay soils with sand in the upper 120 cm within dike ring | Non-calcareous sabulous clay soil |
| 83 | 13 | Non-calcareous sabulous and light clay soils rich in iron with sand | Non-calcareous sabulous clay soil |
| 84 | 13 | Non-calcareous sabulous and light clay soils with sand in the upper 120 cm within dike ring | Non-calcareous sabulous clay soil |
| 85 | 13 | Non-calcareous sabulous and light clay soils rich in iron with sand in the upper 120 cm within dike ring | Non-calcareous sabulous clay soil |
| 89 | 2 | Calcareous riverine sandy soils with clay cover | Calcareous sandy soil |
| 90 | 4 | Non-calcareous riverine sandy soils with clay cover | Non-calcareous sandy soil |
| 91 | 10 | Brook or riverine clay fed by weak local seepage | Calcareous clay soil |
| 92 | 10 | Brook or riverine clay fed by weak local seepage, topsoil rich in iron | Calcareous clay soil |
| 93 | 10 | Calcareous ‘bowl soils’ on peat soil | Calcareous clay soil |
| 94 | 12 | Non-calcareous ‘bowl soils’ on peat soil | Non-calcareous clay soil |
| 95 | 10 | Clay soil on sunken peat soil | Calcareous clay soil |
| 96 | 10 | Mound | Marine clay soil |
| 97 | 10 | Former agricultural land with clay or sabulous clay topsoil | Calcareous clay soil |
| 98 | 8 | Forest and brook peat soils rich in nutrients and bases | Peat soil |
| 99 | 8 | Forest and brook peat soils rich in nutrients, bases and iron | Peat soil |
| 101 | 10 | Terrace or slope with shallow lime-weathering | Calcareous clay soil |
| 102 | 6 | Calcareous moist to wet marine clay soil | Marine clay soil |
| 103 | 6 | Non-calcareous moist to wet marine clay soil | Marine clay soil |
| 104 | 10 | Silty clay soils | Calcareous clay soil |
| 105 | 6 | Salt marshes with walls and ditches | Marsh soil |
| 106 | 18 | Occasionally flooded alluvium | Marine alluvium |
| 107 | 2 | Green beaches | Calcareous sandy soil |
| 108 | 12 | Old river clay soils, moderately base poor | Non-calcareous clay soil |
| 109 | 12 | Old river clay soils, moderately base poor, rich in iron | Non-calcareous clay soil |
| 111 | 8 | Eutrophic, moderately base-rich peat soil | Peat soil |
| 112 | 8 | Eutrophic, moderately base-rich peat soil, rich in iron | Peat soil |
| 117 | 8 | Oligotrophic, acidic peat soil | Peat soil |
| 119 | 8 | Peat soil with moderate regional seepage | Peat soil |
| 120 | 8 | Peat soil with moderate regional seepage, rich in iron | Peat soil |
| 121 | 8 | Freshwater/slightly brackish terrestrialization peat | Peat soil |
| 129 | 7 | Boggy soil on sand, without seepage | Boggy soil |
| 130 | 7 | Boggy soil on sand, with strong seepage | Boggy soil |
| 131 | 7 | Boggy soil on sand, with strong seepage, rich in iron | Boggy soil |
| 133 | 7 | Boggy dune valley | Boggy soil |
| 134 | 9 | Freshwater dune marsh | Marsh soil |
| 135 | 9 | Young, brackish dune marshes | Marsh soil |
| 136 | 9 | Wet low-lying area (brooks) | Calcareous clay soil |
| 138 | 2 | Lime walls | Calcareous sandy soil |
| 139 | 5 | Dike | Dikes and levees |
| 140 | 3 | Water | Water |
| 141 | 1 | Not assessed | No data |
| 142 | 1 | No solution | No data |
| 143 | 1 | Not assessed | No data |
| 144 | 1 | No data | No data |

**Table S4 | Number of occurrence records for each of the reptile species in the case-study municipalities. For the model evaluation, we selected species-municipality combinations with at least 10 occurrence records.**

| **Municipality** | **Species** | **Number of records** |
| --- | --- | --- |
| Deurne | Smooth snake (*Coronella austriaca*) | 39 |
| Deurne | Viviparous lizard (*Zootoca vivipara*) | 77 |
| Deurne | Sand lizard (*Lacerta agilis*) | 53 |
| Ede | European Viper (*Vipera berus*) | 182 |
| Ede | Smooth snake (*Coronella austriaca*) | 350 |
| Ede | Slow worm (*Anguis fragilis*) | 1179 |
| Ede | Viviparous lizard (*Zootoca vivipara*) | 239 |
| Ede | Grass snake (*Natrix helvetica*) | 206 |
| Ede | Sand lizard (*Lacerta agilis*) | 4857 |
| Epe | European Viper (*Vipera berus*) | 267 |
| Epe | Smooth snake (*Coronella austriaca*) | 169 |
| Epe | Slow worm (*Anguis fragilis*) | 373 |
| Epe | Viviparous lizard (*Zootoca vivipara*) | 541 |
| Epe | Grass snake (*Natrix helvetica*) | 121 |
| Epe | Sand lizard (*Lacerta agilis*) | 769 |
| Horst aan de Maas | Smooth snake (*Coronella austriaca*) | 53 |
| Horst aan de Maas | Viviparous lizard (*Zootoca vivipara*) | 85 |
| Huizen | Viviparous lizard (*Zootoca vivipara*) | 163 |
| Huizen | Grass snake (*Natrix helvetica*) | 16 |
| Huizen | Sand lizard (*Lacerta agilis*) | 134 |
| Ommen | European viper (*Vipera berus*) | 105 |
| Ommen | Slow worm (*Anguis fragilis*) | 32 |
| Ommen | Viviparous lizard (*Zootoca vivipara*) | 227 |
| Ommen | Grass snake (*Natrix helvetica*) | 113 |
| Ommen | Sand lizard (*Lacerta agilis*) | 444 |
| Roermond | European viper (*Vipera berus*) | 1 |
| Roermond | Slow worm (*Anguis fragilis*) | 35 |
| Roermond | Viviparous lizard (*Zootoca vivipara*) | 129 |
| Roermond | Grass snake (*Natrix helvetica*) | 1 |
| Roermond | Sand lizard (*Lacerta agilis*) | 93 |
| Westerveld | European Viper (*Vipera berus*) | 2891 |
| Westerveld | Smooth snake (*Coronella austriaca*) | 325 |
| Westerveld | Slow worm (*Anguis fragilis*) | 636 |
| Westerveld | Viviparous lizard (*Zootoca vivipara*) | 2017 |
| Westerveld | Common wall lizard (*Podarcis muralis*) | 128 |
| Westerveld | Grass snake (*Natrix helvetica*) | 637 |
| Westerveld | Sand lizard (*Lacerta agilis*) | 164 |
| Wormerland | Grass snake (*Natrix helvetica*) | 53 |
| Zutphen | Slow worm (*Anguis fragilis*) | 29 |
| Zutphen | Viviparous lizard (*Zootoca vivipara*) | 1 |
| Zutphen | Grass snake (*Natrix helvetica*) | 17 |

**Table S5** **| Variable importance in ensemble SDM per species.**

| **Variable** | **European viper** | **Smooth snake** | **Slow worm** | **Viviparous lizard** | **Common wall lizard** | **Grass snake** | **Sand lizard** |
| --- | --- | --- | --- | --- | --- | --- | --- |
| ***Climate variables*** | | | | | | | |
| Mean annual temperature | 0.36 | 0.006 | 0.003 | 0.002 | 0.07 | 0.2 | 0.02 |
| Temperature seasonality | 0.008 | 0.03 | 0.006 | 0.0009 | 0.008 | 0.005 | 0.01 |
| Mean annual precipitation | 0.004 | 0.01 | 0.01 | 0.0007 | 0.001 | 0.14 | 0.002 |
| Precipitation seasonality | 0.15 | 0.02 | 0.05 | 0.03 | 0.11 | 0.08 | 0.002 |
| ***Land cover variables*** | | | | | | | |
| Grassland | 0.16 | 0.1 | 0.06 | 0.07 | 0.2 | 0.08 | 0.13 |
| Agriculture | 0.15 | 0.1 | 0.06 | 0.06 | 0.13 | 0.05 | 0.08 |
| Greenhouses | 0.02 | 0.01 | 0.006 | 0.007 | 0.02 | 0.006 | 0.007 |
| Orchards | 0.05 | 0.01 | 0.02 | 0.02 | 0.07 | 0.02 | 0.02 |
| Deciduous forest | 0.10 | 0.04 | 0.04 | 0.04 | 0.15 | 0.12 | 0.05 |
| Coniferous forest | 0.05 | 0.1 | 0.24 | 0.15 | 0.07 | 0.05 | 0.18 |
| Portion fresh water | 0.16 | 0.2 | 0.07 | 0.07 | 0.22 | 0.11 | 0.10 |
| Portion salt water | 0.02 | 0.09 | 0.007 | 0.006 | 0.01 | 0.005 | 0.008 |
| Portion built area | 0.16 | 0.02 | 0.05 | 0.07 | 0.26 | 0.08 | 0.07 |
| Forest in built area | 0.07 | 0.1 | 0.02 | 0.03 | 0.1 | 0.04 | 0.03 |
| Grass in built area | 0.11 | 0.06 | 0.04 | 0.04 | 0.72 | 0.05 | 0.05 |
| Paved area | 0.15 | 0.08 | 0.05 | 0.05 | 0.53 | 0.14 | 0.06 |
| Marsh vegetation | 0.03 | 0.09 | 0.01 | 0.01 | 0.03 | 0.02 | 0.02 |
| Open sands | 0.01 | 0.03 | 0.004 | 0.003 | 0.01 | 0.006 | 0.006 |
| Dunes | 0.008 | 0.01 | 0.002 | 0.002 | 0.005 | 0.002 | 0.004 |
| Heather | 0.05 | 0.006 | 0.005 | 0.05 | 0.01 | 0.01 | 0.03 |
| Moderately grass-encroached heather | 0.008 | 0.13 | 0.005 | 0.005 | 0.01 | 0.01 | 0.01 |
| Strongly grass-encroached heather | 0.006 | 0.03 | 0.004 | 0.004 | 0.01 | 0.01 | 0.005 |
| Raised bog | 0.007 | 0.01 | 0.0004 | 0.02 | 0.001 | 0.002 | 0.0006 |
| Forest in raised bog | 0.007 | 0.06 | 0.0004 | 0.0007 | 0.001 | 0.002 | 0.0006 |
| Reed vegetation | 0.02 | 0.02 | 0.009 | 0.007 | 0.02 | 0.01 | 0.01 |
| Forest in marsh area | 0.02 | 0.02 | 0.007 | 0.005 | 0.02 | 0.02 | 0.009 |
| Coastal grassland | 0.01 | 0.008 | 0.003 | 0.002 | 0.006 | 0.002 | 0.04 |
| Shrub in raised bog | 0.001 | 0.004 | 0.0004 | 0.0007 | 0.001 | 0.002 | 0.0005 |
| Shrub in marsh | 0.02 | 0.02 | 0.009 | 0.006 | 0.02 | 0.02 | 0.01 |
| Other shrub vegetation | 0.06 | 0.08 | 0.02 | 0.02 | 0.1 | 0.12 | 0.03 |
| No data | 0.29 | 0.13 | 0.07 | 0.09 | 0.07 | 0.03 | 0.09 |
| ***Soil variables*** | | | | | | | |
| Soil type | 0.001 | 0.003 | 0.001 | 0.0004 | 0.001 | 0.002 | 0.005 |

**Table S6 | Evaluation scores for the ensemble SDMs at national extent.**

| **Species** | **AUC-score** | **TSS-score** | **Sensitivity** | **Specificity** |
| --- | --- | --- | --- | --- |
| European viper (*Vipera berus*) | 0.99 | 0.94 | 0.98 | 0.97 |
| Smooth snake (*Coronella austriaca*) | 0.99 | 0.92 | 0.95 | 0.97 |
| Slow worm (*Anguis fragilis*) | 0.99 | 0.92 | 0.95 | 0.97 |
| Viviparous lizard (*Zootoca vivipara*) | 0.97 | 0.81 | 0.92 | 0.89 |
| Common wall lizard (*Podarcis muralis*) | 0.99 | 0.90 | 0.97 | 0.86 |
| Grass snake (*Natrix helvetica*) | 0.95 | 0.76 | 0.89 | 0.87 |
| Sand lizard (*Lacerta agilis*) | 0.99 | 0.90 | 0.95 | 0.95 |

**Table S7 | Balanced model accuracy (BMA) per species at national and municipal levels for ensemble SDMs computed in this study.**

| **Species** | **BMA at national level** | **BMA at municipal level** | **n** |
| --- | --- | --- | --- |
| European viper (*Vipera berus*) | 0.97 | 0.79 (SD: 0.11) | 5 |
| Smooth snake (*Coronella austriaca*) | 0.96 | 0.72 (SD: 0.07) | 5 |
| Slow worm (*Anguis fragilis*) | 0.91 | 0.61 (SD: 0.13) | 6 |
| Viviparous lizard (*Zootoca vivipara*) | 0.91 | 0.62 (SD: 0.11) | 9 |
| Common wall lizard (*Podarcis muralis*) | 0.91 | 0.91 (SD: 0.00) | 1 |
| Grass snake (*Natrix helvetica*) | 0.88 | 0.52 (SD: 0.23) | 8 |
| Sand lizard (*Lacerta agilis*) | 0.95 | 0.71 (SD: 0.13) | 7 |

# **Figures**


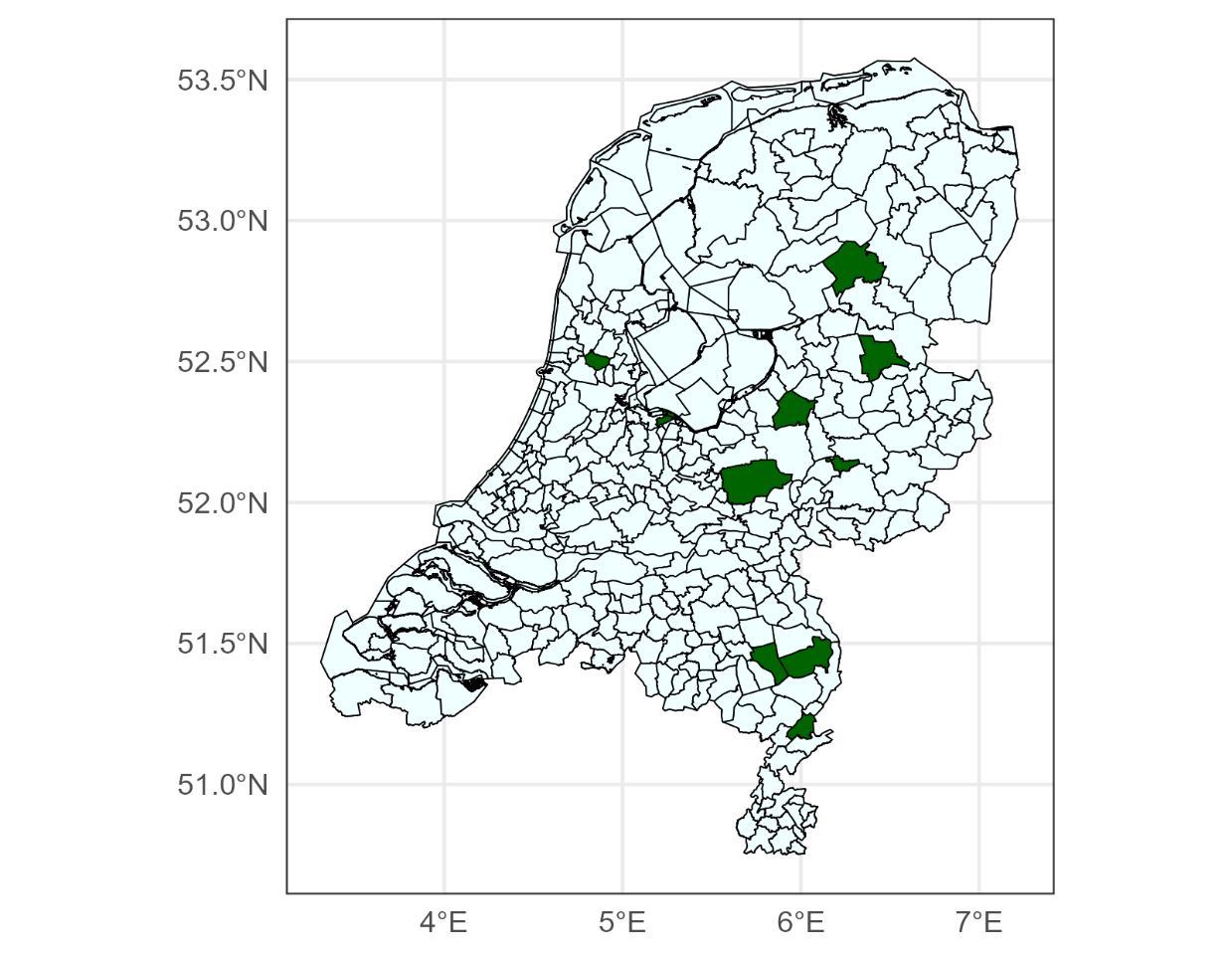


**Figure S1 | Map of municipalities in the Netherlands with test municipalities highlighted (dark green).**

**
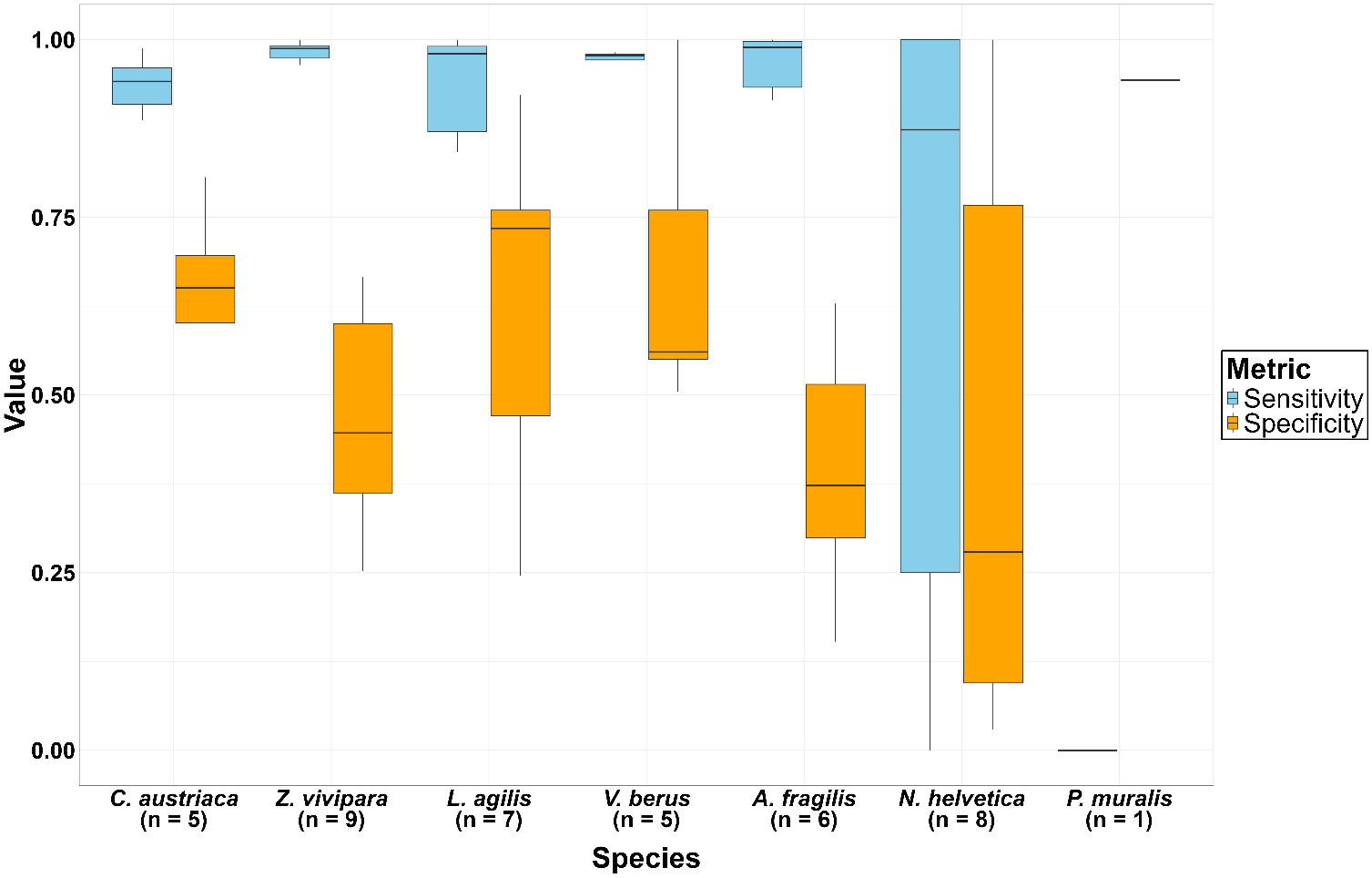
**

**Figure S2** **| Sensitivity and specificity scores at local level per species.** Median values are displayed by the horizontal black line within the boxes showing the interquartile range (IQR) (distance between first and third quantiles of data). Whiskers extend to 1.5 times the IQR-distance from the edge of the upper and lower limits of the box. Sample sizes (x-axis) represent the number of municipalities used in the evaluation (Table S5).


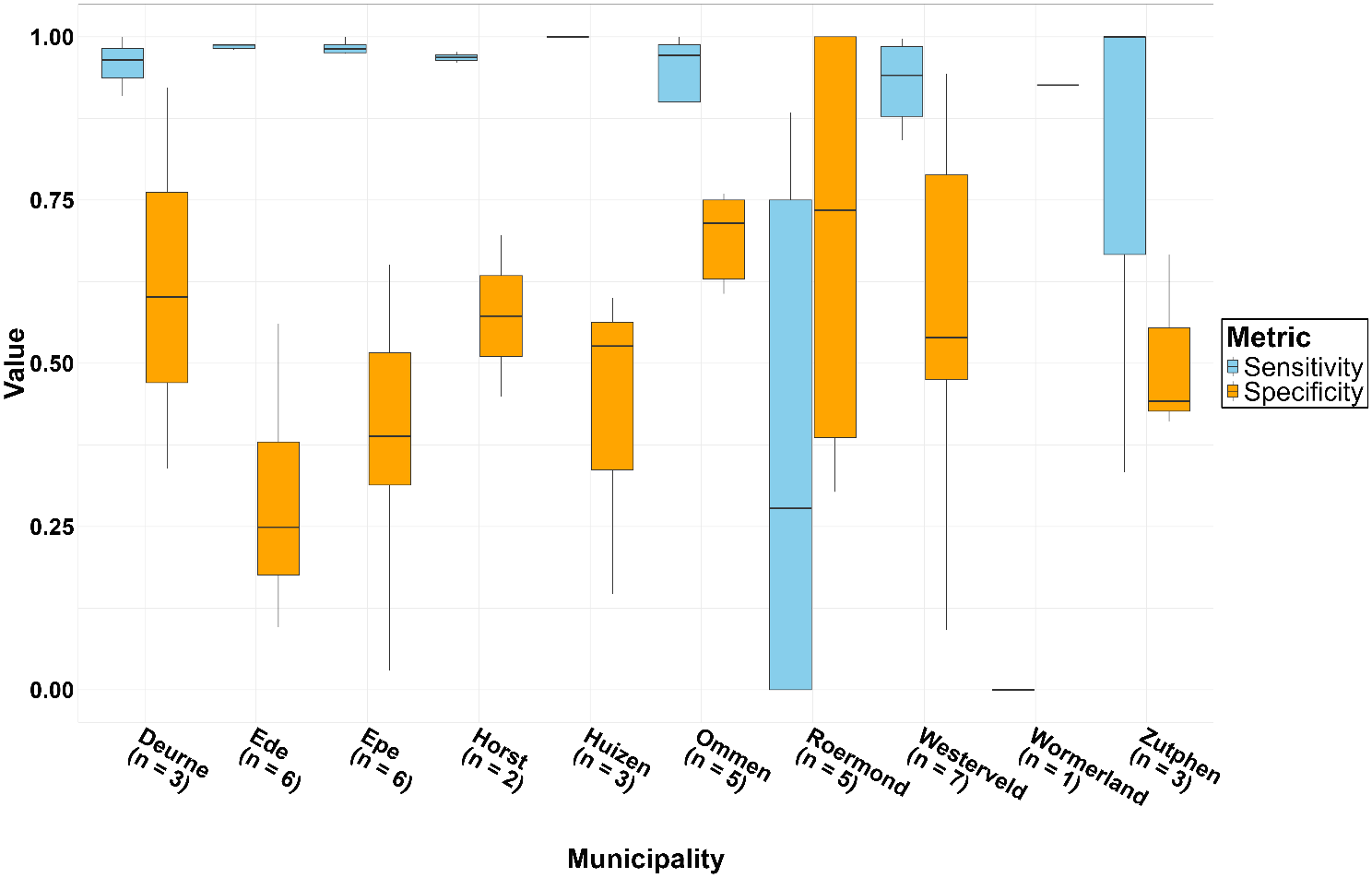


**Figure S3** **| Sensitivity and specificity scores for the 10 test municipalities.** Median values are displayed by the horizontal black line within the boxes showing the interquartile range (IQR) (distance between first and third quantiles of data). Whiskers extend to 1.5 times the IQR-distance from the edge of the upper and lower limits of the box. Sample sizes (x-axis) represent the number of species used in the evaluation (Table S5).


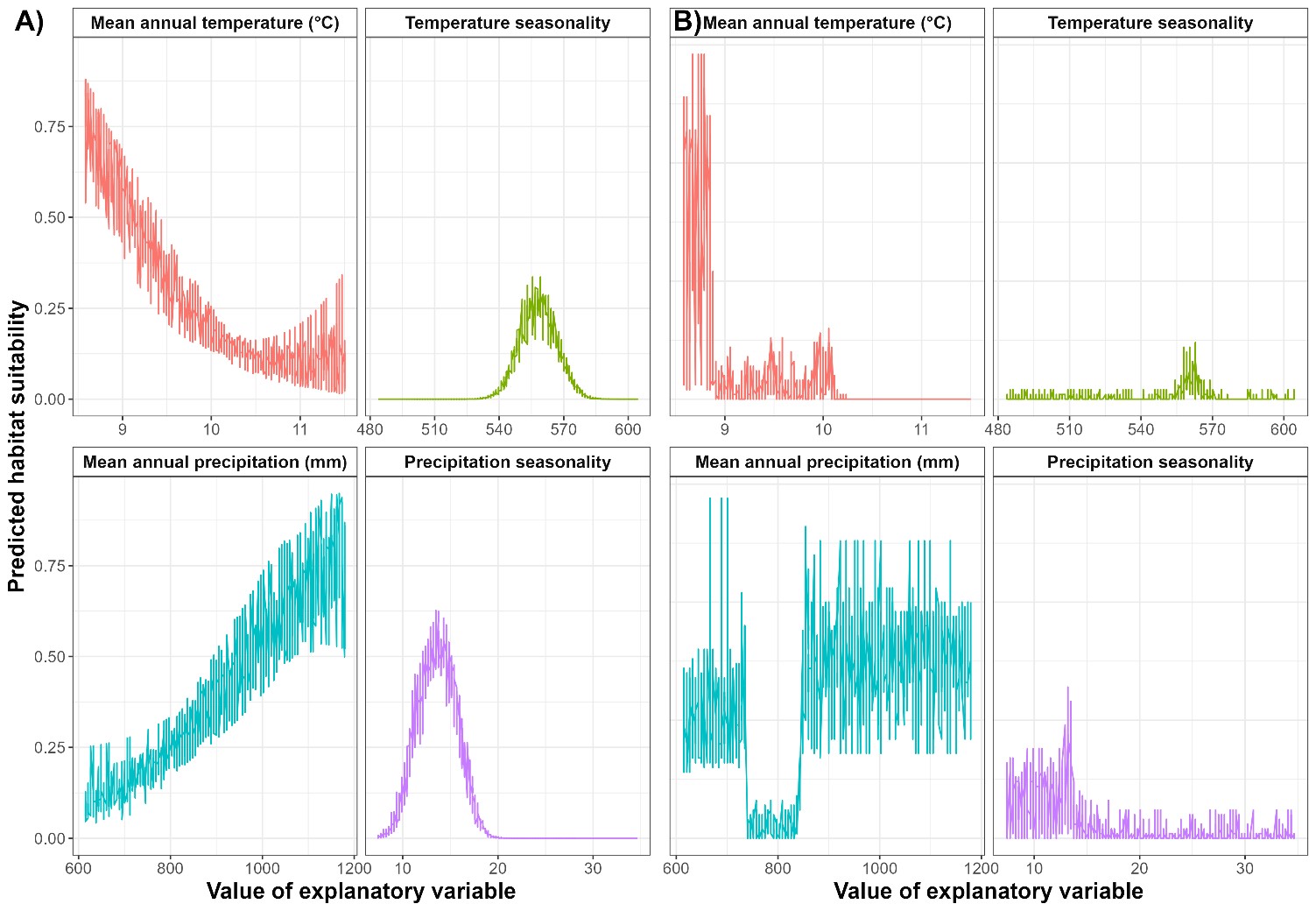


**Figure S4 | Response curves of *C. austriaca* for four climatological variables of A) GLM and B) RF algorithms.** The different variables are displayed in separate panels. The x-axis displays the variables’ values, while the y-axis shows the predicted habitat suitability value based on the value of the environmental variable. Precipitation and temperature seasonality are standardised hence do not have a unit.
